# Videofluoroscopy Practice in India: A Survey of Speech-Language Pathologists

**DOI:** 10.1007/s00455-022-10487-5

**Published:** 2022-07-08

**Authors:** Rahul Krishnamurthy, Bhavana Bhat, Priyanka Suresh Nayak, Radish Kumar Balasubramanium

**Affiliations:** 1 University of Nebraska, Lincoln, USA; 2 Department of Audiology and Speech Language Pathology, Kasturba Medical College, Mangalore, Karnataka India; 3 Manipal Academy of Higher Education, Manipal, India

**Keywords:** Dysphagia, Deglutition, Deglutition disorders, Videofluoroscopy, Clinical practice

## Abstract

Owing to differences in clinical approaches toward videofluoroscopic swallowing studies (VFSS), professional and governing bodies in some countries have implemented guidelines for performing VFSS. However, in India, the establishment of such practice guidelines is in initial stages of acceptance and there are no uniform guidelines. The aim of the current study was to investigate and describe the clinical practice patterns related to VFSS assessments among Speech-Language Pathologists (SLPs) in India. An electronic survey consisting of 34 questions categorized into four main sections (demographic details and education; current practice; instrumental and technical considerations; protocol and assessment methods) was sent to SLPs registered with the Indian Speech and Hearing Association (ISHA) through email and social media from August 2020 to January 2021. A total of 129 eligible responses were received. More than 50% of the participants used a standard assessment and analysis protocol. Barium and water-soluble contrasts were mostly used, and 97% of participants did not know what percent weight to volume (w/v) or volume to volume (v/v) contrast to fluid were used. Considerable amount of variability was observed in the responses of our participants, which was consistent with international surveys of VFSS practice patterns. Lack of adequate radiation safety measures was also observed. Our findings suggest a need to increase awareness and training among Indian SLPs in technical and procedural aspects of VFSS, and more sensitization toward radiation safety. ISHA should consider forming a committee to develop and adopt uniform nationwide practices in VFSS. Future studies exclusively investigating barriers and facilitators to VFSS practice in the Indian scenario are necessary.

**Supplementary Information:**

The online version contains supplementary material available at 10.1007/s00455-022-10487-5.

## Introduction

Videofluoroscopic swallowing studies (VFSS), also known as the modified barium swallow studies (MBSS), are one of the “gold standard” assessments to identify structural and functional causes of dysphagia. Apart from providing information about the anatomy and physiology of the oropharyngeal swallow, VFSS can offer information about the effectiveness of therapeutic techniques and compensatory strategies. Usually, VFSS is performed by speech-language pathologists (SLPs) working alongside imaging professionals. The VFSS procedure is complex and presents the potential for variations in clinical practice. Differences in clinical approaches to VFSS may be due to several factors including, but not limited to variability in education, training, and availability of resources.

Owing to differences in clinical approaches toward VFSS, professional and governing bodies in some countries have implemented clinical practice guidelines for performing VFSS [1, 2]. The need for standard VFSS guidelines has also been emphasized in a recent systematic review by Boaden et al. [3]. In the United Kingdom, the Royal College of Speech and Language Therapy (RCSLT) position paper on VFSS [1] provides guidelines for practicing SLPs. The RCSLTs’ position paper recommends a team consisting of an SLP, and aradiologist/radiographer to perform VFSS. Citing significant variability in the literature and published protocols, RCSLT recommends that clinicians determine appropriate local protocols.

Clinical guidelines of the west are tailored to meet their local conditions. For instance, New Zealand’s clinical guidelines on VFSS [2] provide detailed recommendations in terms of training, safety, team members, assessment procedure, interpretation, and reporting. In terms of radiation safety, the guideline recommends that clinicians adhere to the principle of keeping radiation dose "as low as reasonably achievable", and all SLPs conducting VFSS are required to wear lead aprons and thyroid shields. A low-density barium suspension (20 – 40% w/v) is recommended. Like the RCSLTs’ recommendation, a team consisting of an SLP, and a radiologist/radiographer is recommended by the New Zealand guidelines. As a part of the VFSS assessment procedure, oral parameters at rest and during swallowing of variety of consistencies, oral transit parameters, pharyngeal parameters at rest and during swallowing of variety of consistencies, laryngeal parameters including penetration-aspiration measures, cricopharyngeal, and oesophageal measures are recommended. Upon closely inspecting the United Kingdom [1], and the New Zealand guidelines [2], one can observe that these guidelines are specific to regional service delivery models and are designed to cater to local clinical needs.

Like its global counterparts, it is necessary for India to have its own guidelines, which are based on explicit evidence but specific to the Indian context. However, in India, establishment of such practice guidelines is in the initial stages of acceptance and there are no uniform guidelines. Services related to dysphagia assessment and management in India are developing [4]. As a result, SLPs in India are required to determine their practice protocols or adapt clinical practice guidelines established in other countries. A survey of dysphagia practice in India by Rangarathnam, and Desai [4] also reported considerable variability in practice for dysphagia assessment and management among SLPs in India; it also highlighted a lack of uniformity across all aspects of dysphagia practice including VFSS.

Variability in VFSS practice can result in quality, safety, and efficiency issues [5]. Differences in clinical practices can result in increased study-to-study variability, and the need to repeat VFSS studies. These variabilities can be minimized by adapting uniform practice guidelines. However, the development and implementation of such uniform clinical guidelines require an understanding of current practice, which have not been comprehensively studied in the Indian context. Thus, the present study aimed to investigate and describe the clinical practice patterns related to VFSS assessments among SLPs in India, which could potentially inform future directions in dysphagia education, clinical training, and developing nation-wide uniform clinical practices.

## Method

### Study Design

We adopted a cross-sectional design with non-randomized convenience sampling. The study protocol was reviewed and approved by the institutional ethical review board of the first author’s institution at the time. The study was conducted at the Department of Audiology and Speech-Language Pathology, Kasturba Medical College, Mangalore, between the years of 2019 and 2021.

### Survey Questionnaire

We began by constructing a survey questionnaire designed to gain insights into Indian SLPs’ VFSS related clinical practice. The initial version of the questionnaire was based on existing western clinical practice studies in VFSS [6, 7]. The questionnaire was content validated by a panel of five SLPs (three practicing clinicians and two academics), with at least five years of clinical experience in dysphagia. The panel members discussed the structure, clarity, ease of administration, and relevance of the questionnaire contents in a round-robin fashion. The content validity of each item on the questionnaire was iteratively refined through feedback cycles. Based on this feedback, minor modifications were made to the questionnaire to improve its understanding and ease of responding to questions. The final electronic version of the survey (Appendix—1) consisted of thirty-four open-ended and close-ended questions categorized into four main sections (demographic details and education; current practice; instrumental and technical considerations; protocol and assessment methods). For each close-ended (multiple choice) question participants could elaborate on their response using open comment boxes throughout the survey.

### Participants

The participants of the survey were SLPs (clinical or academic) who were currently practicing in India. To participate in the survey, SLPs were required to be involved in dysphagia assessment at the time of the survey. All the participants had a minimum qualification of a bachelor’s degree in Audiology and Speech-Language Pathology (BASLP) from a university recognized by the University Grants Commission (UGC) of India, and the Rehabilitation Council of India (RCI).

### Survey

The electronic version of the questionnaire was sent out to all SLPs registered with the Indian Speech and Hearing Association (ISHA), as well as to several local state associations and educational institutions in India. Additionally, the questionnaire was circulated to professional (SLP) groups on social media such as Twitter, and WhatsApp. The online survey remained accessible for six months (August 2020–January 2021). The participants were required to complete all the questions of the survey and were provided with a two-week time limit for completion.

### Data Analysis

The survey results were exported into a Microsoft Excel file and were analyzed using descriptive methods (number of respondents and percentage).

## Results

### Survey Response

A total of 218 anonymous responses were received, and among these 89 participants were not currently involved in swallowing assessment at their workplace. Hence, their responses were discarded and only 129 responses were descriptively analyzed. The average time taken by the participants to complete the survey was 17.56 min.

### Participant Demographics

Demographic details of the participants are shown in Table 1. Among 129 responses from eligible participants, 48 (36%) participants reported that they held a bachelor’s degree in audiology and speech-language pathology, 70 (55%) participants had a master’s degree, and 11 (8%) participants had a doctoral degree or higher. Most of our participants (65%) worked at a medical facility, and most (48%) had less than two years of work experience.

**Table 1:** Demographic details of the participants

Work settings	Number (%)
Medical facility	84 (65%)
University/educational institution	35 (27%)
Private clinic	10 (8%)
*Years of experience*	
Less than two years	62 (48%)
3–5 years	43 (33%)
6–10 years	13 (11%)
More than 10 years	11 (8%)

### Assessment Practice

#### Caseload and Referrals

A substantial proportion (36%) of our participants performed three to five swallowing assessments per week. Table 2 shows the number of swallowing assessments performed per week by our participants.

**Table 2:** Number of swallowing assessments performed per week

Swallowing assessments performed per week	Number (%)
Less than two	35 (27%)
3–5	46 (36%)
6–10	18 (14%)
More than 10	30 (23%)

We followed up by asking the participants about their sources of referrals for swallowing assessment. Forty-nine participants (38%) reported that they received majority of the referrals for swallowing assessment came from an otorhinolaryngologist, 22 participants (17%) revealed their source to be a general physician, 39 participants (30%) reported that they received most of their referrals from a neurosurgeon/ neurologist, and 19 participants (15%) reported other professionals, which included pediatricians and gastroenterologists.

Around 60% of our participants performed less than two VFSS assessments per week. Table 3 shows the number of VFSS assessments performed per week by our participants. We were also interested in knowing the basis did our participants recommend VFSS assessments. Suspected silent aspiration was the common most criteria (42%) reported by our participants for recommending VFSS. Table 4 shows other referral criterion reported by our participants.

**Table 3:** Number of VFSS assessments performed per week

VFSS assessments performed per week	Number (%)
Less than two	77 (60%)
3 – 5	47 (36%)
6 – 10	5 (4%)
More than 10	0

**Table 4:** Referral criteria for VFSS assessment

Referral criteria	Number (%)
Fail on a standardized swallowing screening test	28 (22%)
Suspected silent aspiration	55 (42%)
Fail on a clinical swallowing examination	32 (25%)
All patients	14 (11%)

#### Assessment Protocols

Seventy-three participants (56%) reported that they used a standard assessment protocol for VFSS at their workplace. Fifty-six participants (44%) did not respond or did not wish to disclose. Among the 73 participants who responded, 62 participants (85%) reported that they used an “in-house” protocol determined by their employer or the current workplace as opposed to protocols taught during their clinical/academic training program. However, none of these participants disclosed what formed their in-house protocol. Seven participants (10%) reported using Logemann’s protocol [8], and four participants (5%) reported using Modified Barium Swallow Impairment (MBSImP) [9] protocol.

#### Test Materials

Forty-three participants (33%) reported testing for liquids only, 59 participants (46%) reported testing both liquids, and solid consistencies, and 27 participants (21%) reported using liquids, semisolids, and solid consistencies. All our participants (100%) were aware of the International Dysphagia Diet Standardisation Initiative (IDDSI) [10] terminologies, however, only 14 participants (18%) modified consistencies based on IDDSI for VFSS testing.

#### Contrast Material

Fifty-seven participants (44%) reported using only liquid barium, 44 participants (34%) used barium powder, and 22 participants (17%) used both suspensions. Six participants (5%) reported that they used gastrografin. Among the 123 participants (95%) who used barium as an agent, 120 participants (97%) did not know what percent weight to volume (w/v) or volume to volume (v/v) contrast to fluid they used. Three participants (less than 3%) gave unclear or unreliable responses.

#### Screening for Esophageal Dysphagia

None of our participants screened for esophageal dysphagia during the VFSS. When we asked our participants the reason to not screen for esophageal dysphagia, 102 participants (79%) reported that it was not within their scope of practice, 17 participants (13%) reported that they were not trained for esophageal screening, and the remaining ten participants (8%) did not respond to the question.

#### VFSS Analysis Method

##### Staff

Seventy-three participants (56%) reported that they analyzed VFSS recordings together with a radiologist/radiographer. The remaining 56 participants (44%) reported that the radiologist alone analyzed VFSS recordings at their workplace.

### Analysis Protocol

A standard protocol was used by 73 participants (56%) to analyze VFSS recordings. Among the 73 participants who used a standard protocol, 63 participants (85%) reported that they used an in-house protocol, six participants (10%) reported the use of Logemann’s protocol [8], and four participants (5%) reported using the Modified Barium Swallow Impairment (MBSImP) [9]. In the cases where analysis was performed by the radiologist, participants were not aware of analysis methods used. Seventy-three participants (56%) reported using the Penetration Aspiration Scale (PAS) [11], and the remaining 56 participants (44%) did not use a rating scale.

#### VFSS Instrumentation and Acquisition

Table 5 shows the participant’s awareness of VFSS instrumentations and its technical aspects. Eighty-seven participants (68%) participants were unaware of the type of video fluoroscopy instrument at their facility. Among the remaining participants (32%), a flat panel detector was used by 30 participants (72%), and an image intensifier system was used by 12 participants (28%). Eighty-three participants (64%) reported that they were aware of the image contrast and brightness settings of the VFSS instrument at their facility. Ninety-five participants (74%) participants were unaware of the imaging modes of the fluoroscopy. Among the remaining 34 participants (26%), 30 participants (88%) reported that they used continuous fluoroscopy, and four participants (12%) reported using a pulsed type of fluoroscopy. One hundred and three participants (71%) reported that they were aware of the frame rate of fluoroscopy instrument at their facility. All participants (100%) reported that they were aware of radiation safety measures during VFSS procedure. However, only 13 participants (10%) reported the use of lead collars and suits, and seven participants (5%) reported the use of lead collars only.

**Table 5:** Awareness of *VFSS instrumentation and technical aspects*

Technical aspect	Number of participants aware (%)
Type of fluoroscopy instrument	42 (32%)
Image contrast and brightness setting	83 (64%)
Imaging mode	34 (26%)
Frame rate	103 (71%)
Radiation safety measure	129 (100%)

## Discussion

Speech-language pathology services, particularly as they relate to assessment and management of dysphagia are still developing and expanding in India [4]. Though VFSS is considered a gold standard tool in the evaluation of dysphagia, there exists variations in the technical specifications and clinical practice patterns among Indian SLPs suggesting the need for standardization of VFSS practice in India for the benefit of patients. Standardization of instrumental dysphagia assessment can lead to, but not limited to, measurable and evidence-based practice, increased clinical confidence and reliability, and improved compliance with practice and reporting, and thereby, improving the patient outcomes, patient safety, and the quality of health care. For this, understanding the current VFSS practice pattern is the first step toward standardization of uniform practice guidelines.

More than 50% of our participants reported using standard protocols for assessment, the majority of these being developed in house. In India, currently there are no structured frameworks/ guidelines on VFSS; for this reason, it is expected that the practicing clinicians develop their own assessment protocols, or the ones determined by their employer/ workplace. Even though a handful of published protocols are available, less than 15% of our participants reported using them in their clinical practice. We believe that these variabilities stem from lack of training, and clinicians’ knowledge of current evidence-based practice standards, which has also been reported to be frequently perceived as a barrier for implementing evidence-based VFSS guidelines [12].

Our participants reported using an array of textures during VFSS assessments. However, around 33% of our participants reported testing a single type of stimulus (only liquids). Best practice protocols from western countries emphasize the need to evaluate swallowing with a variety of consistencies and volumes [1, 2, 9]. When performing VFSS, it may not be necessary to test all types of textures and volumes, doing so has been reported to contradict the very principal goal of VFSS [5]. Rather, the textures and volumes tested should be decided based on reason for requesting VFSS, the findings in clinical swallowing examination, and the patient’s performance during the procedure. Recent best evidence has demonstrated that by using a standardized set of consistencies in conjunction with the MBSImp [9] protocol can best identify the presence and nature of swallowing impairment [13].

For fluids and foods to be visualized during the VFSS procedure, addition of a radiopaque substance is needed. Nearly, all our participants (95%) reported using barium sulfate as contrast material for VFSS assessments, which is similar to western reports [6, 7]. However, the addition of barium sulfate to fluids used during VFSS changes the density and viscosity of the original material [14–16]. It is possible to achieve correct viscosities by using standardized mixing and testing protocols, such as the IDDSI testing procedures. However, the majority of our participants (82%) reported the use of non-standardized recipes during VFSS assessments; this suggests that in clinical practice, variability may exist in the texture of oral trials and contrast concentration. These results also reflect the knowledge and practice of our participants about the IDDSI terminologies and testing methods. Even though all our participants were aware of IDDSI terminologies and testing methods, only 18% of them reported using IDDSI method. This may be due to factors such as lack of training, time constraints, and lack of facility. A study by Lemire, Miles, and McCann [12] has also acknowledged time constraint and resources as a barrier for implementing evidence-based VFSS guidelines.

Recipes also require standardizing the concentration of contrast used to ensure adequate visibility on images without them leaving a coating in the oral or pharyngeal cavities [17, 18]. Any concentration greater than 40% weight to volume (w/v) of barium sulfate is reported to leave behind a coating [17] and may be misinterpreted as residue due to pharyngeal stage impairment. Surprisingly, 97% of our participants were not aware of the percent weight to volume (w/v) or volume to volume (v/v) contrast to fluid they used. Similar findings been reported by western survey reports [6, 7].

The American Speech-Language Hearing Association (ASHA) [19] recommends evaluation of all phases of swallowing during assessment; other studies [20–22] also recommend the same. Screening for esophageal dysphagia during a swallow assessment is well within the SLP scope of practice [19, 20] and is gaining ground. However, in the current study, none of our participants performed esophageal screening during VFSS assessment, citing the procedure to be out of scope and due to lack of training. There is a need to increase awareness, and training provided to Indian SLPs to promote the integration of esophageal screening into VFSS examination.

The successful interpretation of VFSS recordings depends on a number of factors, including, the quality of the images obtained, the clinician’s skills (education, experience, and confidence), and on human visual perception and the ability to recognize patterns. Studies [23, 24] have reported that the reliability of VFSS analysis and interpretation could be improved using standard protocols, and rating scales. The results of the current study suggest that the analysis of VFSS recordings may be radiologist led in the Indian context suggesting the strong need for advocacy with regard to the SLP scope of practice in conducting and interpreting VFSS in India. Since it is within the SLP scope of practice to conduct VFSS, SLPs should be one of the primary team members and receive the appropriate clinical training to implement this change in practice.

Only 56% of our participants reported using a rating scale to analyze VFSS recordings, and the PAS [11] was reported to be used by all these participants. Interestingly, the participants, who reported using PAS also reported that they analyzed VFSS recordings alone or with a radiographer/ radiologist. For those participants (44%), who did not use rating scales, the VFSS analyses were performed by a radiographer/ radiologist alone. These participants were unaware of rating scales used by their radiographer/ radiologist to analyze VFSS recordings. The PAS [11] appears to be the most widely used rating scale to grade the severity of dysphagia as seen on VFSS recordings. Similar findings have been reported in a survey, which investigated VFSS practice patterns in the United Kingdom [6]. It was observed that none of our participants used standardized validated tools such as MBSImP [9], or Analysis of Swallowing Physiology: Events, Kinematics and Timing (ASPEKT) [25] for VFSS interpretation. It may be possible that these participants were either completely unaware of these analysis protocols or lacked training in using these protocols. However, the survey did not collect any information on the knowledge of using these analysis protocols in the present study.

The PAS was originally designed to describe and quantify severity of penetration and aspiration events during swallowing. The scale has been extensively used by both dysphagia clinicians and researchers across the globe over 20 years. As acknowledged by the original authors [11], the scale deals only with the depth of airway invasion or whether the material is cleared (ejected) to a safer location. The scale does not capture the mechanisms responsible for unsafe swallows such as timing of airway invasion, premature bolus spillage, or the post swallow residue. Emergent literature [26, 27] has also questioned its statistical constructs and its application in research. We caution clinicians to be aware of these pitfalls; interested readers can refer to the work by Steele, and Grace-Martin [26], which provides a detailed critique on the statistical construct of the PAS and its clinical use. The current survey did not probe into how VFSS findings are reported. Future studies should consider the inclusion of report writing.

A quality VFSS recording requires understanding of technical features of videofluorosocpy, such as equipment type, image contrast, imaging modes, imaging resolution, and safety considerations. In the current study, 68% of our participants were unaware of the type of video fluoroscopy, image contrast and brightness setting (26%), imaging mode (74%), and frame rate (19%). Usually, Indian SLPs are trained in-house with respect to VFSS; the limited knowledge of Indian SLPs regarding technical aspects of VFSS reflects the in-house training, which may not cover information on technical aspects of VFSS. It may also reflect limited communication between SLPs and VFSS technical personnel such as radiographers. A similar trend was reported among practitioners in the UK, where a high percentage of respondents were not aware of technical features of VFSS [4].

Even though all the participants reported awareness of radiation safety measures during VFSS, only 15% of the participants reported the use of lead collars and aprons. The reason for 85% of participants failing to follow the radiation safety measures were not further probed in the survey, and we acknowledge this to be a limitation. However, it may be possible that majority of the SLPs are at the analysis end during the entire procedure as 44% responded that the radiologist alone analyzed the VFSS recordings at their respective facilities. Nevertheless, there is a need to increase awareness and sensitize Indian SLPs toward radiation safety. Time, distance, and shielding are the three main factors to be considered for safe radiation practices [28]. To minimize radiation exposure, both to the patient and the clinician, the amount of time for which the radiation is on should be functionally minimized. Clinicians should use available personal protective equipment, such as, lead aprons, and thyroid shields. The use of lead aprons and thyroid shields has been reported to reduce radiation exposure by absorbing about 95% of scattered radiation [29]. We recommend interested readers to refer work by Ingleby, Bonhila, and Steele [30] for a detailed tutorial on radiation risk, and safety procedures that clinicians can take while performing VFSS.

## Limitations and Future Directions

Dysphagia practice related services are still growing in India. Taking this factor into account, we designed our survey to investigate knowledge and practice related aspects. However, patterns in reporting VFSS findings were omitted. We could observe that the majority of our participants had less than two years of experience, and the survey did not collect information on geographical location (urban versus rural) of the participants. As with any web-based survey, there is an uncertainty whether the responses reflected true practice or knowledge of the participants. We circulated the survey through various social media channels; consequently, it was not possible to determine the true response rate.

We were also unable to rule out the possibility of multiple responses from individuals working in the same medical facility/ university, which could have skewed the responses. Only descriptive methods (percentage and number of respondents) were used to report the survey data. Prospective studies involving qualitative interviews regarding barriers and facilitators to VFSS practice in India are in progress It may also be necessary to investigate VFSS related knowledge and practice patterns of medical professionals (such as radiologists) to gain additional insights.

## Conclusion

The current survey explored VFSS related knowledge and practices of SLPs in India. We observed variable VFSS practice patterns across all stages of the VFSS procedure. This lack of uniform standards can hinder the ability to make valid comparisons, and negatively impact patient care across health care settings. It is crucial for Indian SLPs to develop and adopt standards for VFSS, for optimal patient care quality, safety, and efficacy. Indian SLPs should collectively consider formation of a dedicated dysphagia-focused group or national body to advocate for uniform national standards in dysphagia assessment and management.

As in many developing nations, there appears to be barriers to access training, and instrumentation. Our findings suggest a need to increase awareness and training among Indian SLPs in technical aspects of videofluoroscopy, performing and analyzing VFSS assessments, and radiation safety measures. Most of our participants reported limited expertise, which translates to reduced opportunities in clinical training. Even though prospective SLPs are trained in VFSS assessments as a part of an educational program, the training program itself will have to incorporate a more thorough standard with specific utilization of recent best practices.

National governing bodies such as the Indian speech and hearing association (ISHA), and the Rehabilitation council of India (RCI) should promote training opportunities in form of continuing rehabilitation education (CRE) programs specifically designed toward VFSS assessments, analysis, and interpretation. Our participants also suggested exclusive inclusion of VFSS in academic curricula. Efforts by these bodies in collaboration with educational institutes, and the government are necessary in developing, and implementing clinical guidelines related to VFSS assessments in India.

### Recommended Readings on Best Practices in VFSS

See Table 6

**Table 6:** List of recommended readings on best practices in VFSS

Standardized protocol	Martin-Harris, B., Brodsky, M. B., Michel, Y., Castell, D. O., Schleicher, M., Sandidge, J., … Blair, J. (2008). MBS Measurement Tool for Swallow Impairment—MBSImp: Establishing a Standard. Dysphagia, 23(4), 392–405. https://doi.org/10.1007/s00455-008-9185-9 Steele, C., Martin, B., Gosa, M., & Allen, S. (2021). Diagnosis and Management of Swallowing Physiology: Standardized Contrast, the MBSImPTM, & the IDDSI Framework. Applied Radiology
Testing material	Cichero, J. A. Y., Lam, P., Steele, C. M., Hanson, B., Chen, J., Dantas, R. O., … Stanschus, S. (2016). Development of International Terminology and Definitions for Texture-Modified Foods and Thickened Fluids Used in Dysphagia Management: The IDDSI Framework. Dysphagia, 32(2), 293–314. https://doi.org/10.1007/s00455-016-9758-y
	International Dysphagia Diet Standardisation Initiative. https://iddsi.org
Contrast material	Steele, C.M., Molfenter, S.M., Péladeau-Pigeon, M. et al. Challenges in Preparing Contrast Media for Videofluoroscopy. Dysphagia 28, 464–467 (2013). https://doi.org/10.1007/s00455-013-9476-7 Swallowing Rehabilitation Research Lab. Barium Calculator: http://steeleswallowinglab.ca/srrl/best-practice/barium-recipes/iddsi-barium-calculator/
Screening for oesophageal dysphagia	Watts, S., Gaziano, J., Jacobs, J., & Richter, J. (2019). Improving the diagnostic capability of the modified barium swallow study through standardization of an esophageal sweep protocol. Dysphagia, 34, 34–42. https://doi.org/10.1007/s00455-018-09966-5
VFSS rating scale	Steele, C.M., Grace-Martin, K. Reflections on Clinical and Statistical Use of the Penetration-Aspiration Scale. Dysphagia 32, 601–616 (2017). https://doi.org/10.1007/s00455-017-9809-z
	Borders, J. C., & Brates, D. (2020). Use of the penetration-aspiration scale in dysphagia research: a systematic review. Dysphagia, 35(4), 583–597
VFSS instrumentation and acquisition	Peladeau-Pigeon, M., & Steele, C. M. (2013). Technical aspects of a videofluoroscopic swallowing study. Canadian Journal of Speech-Language Pathology and Audiology, 37(3), 216–226
	Bonilha, H.S., Blair, J., Carnes, B. et al. Preliminary Investigation of the Effect of Pulse Rate on Judgments of Swallowing Impairment and Treatment Recommendations. Dysphagia 28, 528–538 (2013). https://doi.org/10.1007/s00455-013-9463-z
Radiation safety	Ingleby, H. R., Bonilha, H. S., & Steele, C. M. (2021). A Tutorial on Diagnostic Benefit and Radiation Risk in Videofluoroscopic Swallowing Studies. Dysphagia, 1–26

## Supplementary Information

Below is the link to the electronic supplementary material.

Supplementary file1 (DOCX 25 KB)
