# Supplementary material for: Videofluoroscopy Practice in India: A Survey of Speech-Language Pathologists
Source: Dysphagia. 2022 Jul 8;38(1):457–65. doi: 10.1007/s00455-022-10487-5 (PMC9873755; doi:10.1007/s00455-022-10487-5)
Supplement: Supplementary file 1 — Supplementary file1 (DOCX 25 KB) [file 455_2022_10487_MOESM1_ESM.docx]

**Appendix 1**

Dear fellow professionals,

You are being invited to take part in a research study conducted by Mr. Rahul Krishnamurthy, and Dr. Radish Kumar from the Department of Audiology and Speech Language Pathology, Kasturba Medical College (MAHE), Mangalore. Before you decide whether to participate, it is important for you to understand why the research is being done and what it will involve. Please take time to read the following information carefully. You are being asked to participate in this research study because you are meeting the selection criteria. The online survey would require approximately 10-15 minutes for you to fill in.

**Why is this study being done?**

The purpose of the online survey is to assess current speech-language pathologists’ (SLPs) practices regarding videofluoroscopy swallow study (VFSS) in India. Even though, there is no immediate direct benefit to you for your participation in this research study, we believe that the findings can aid us in setting up evidence based VFSS clinical practices in India.

**What about confidentiality?**

Information from this study including your name, address, study results will be reviewed only by authorized personnel or representatives, ethics committee or regulatory bodies who will be responsible for doing this research and drawing proper inferences and conclusions. Information and results from the study may be presented at meetings or published in journals without including your name and other personal identifications.

**What Are My Rights?**

Your participation in this research study is voluntary. You may choose not to be in the study. If you agree to be in the study, you may withdraw from the study at any time. If you withdraw from the study, no new data about you will be collected for research purposes.

**What are the benefits of participation in the study?**

Although your participation will be of no direct benefit to you, it may help us to better understand current clinical practices related VFSS in India. The survey information will allow us to infer on challenges and resources available and can aid us setting up evidence based clinical practices in India. You can access free international webinars, research papers and resources for SLPs hosted by the Department of Audiology and Speech Language Pathology, Kasturba Medical College (MAHE) on Twitter (@ASLPKMCMLR). Please indicate that you were referred via this survey.

**VFSS practice pattern in India – a survey**

1. Consent - Please select 'YES' to continue or 'NO' to discontinue.

- Yes
- No

**Demographics and education**

1. Do you perform VFSS at your setup?

- Yes
- No

1. What is your educational qualification?

- Under graduation
- Post graduation
- Doctoral degree (Ph.D)
- Others, Please specify _________

1. How many years of professional experience do you have?

- less than two years
- 3 – 5 years
- 6-10 years
- more than 10 years

1. What type of a setup do you work in?

- Medical facility (Hospital, Nursing home)
- University/ educational teaching facility
- Private practice
- Others, Please specify _________

**Current practice**

1. Are you involved in swallowing assessment?

- Yes
- No

1. On an average, how many dysphagia cases do you see in a week?

- less than two swallowing assessments
- 3 – 5 swallowing assessments
- 6 - 10 swallowing assessments
- more than 10 swallowing assessments

1. What are the sources of your dysphagia assessment referrals?

- ENT/Otorhinolaryngologist
- General physician
- Neurologist/neurosurgeon
- Others, Please specify _________

1. Do you perform VFSS at your facility?

- Yes
- No

1. On an average, how many VFSS you perform in a week?

- less than two VFSS assessments
- 3 – 5 VFSS assessments
- 6 - 10 VFSS assessments
- more than 10 VFSS assessments

1. When do you refer a patient for VFSS examination?

- All patients undergo VFSS assessment
- Patients who fail initial swallowing screening undergo VFSS assessment
- Only those patients suspected of aspiration during clinical swallowing examination undergo VFSS assessment
- Only those patients suspected of silent aspiration undergo VFSS assessment
- Others, Please specify _________

**Instrumental and technical considerations**

1. Do you perform VFSS at your facility?

- Yes
- No

1. Are you aware of type of fluoroscopy equipment used at your facility?

- Yes
- No

1. If Yes, please select the appropriate fluoroscopy equipment type used at your facility.

- Flat panel detector
- Image intensifier
- Others, Please specify _________

1. Are you aware of image contrast and brightness settings of VFSS recording system used at your facility?

- Yes
- No

1. Are you aware of imaging mode of VFSS recording system used at your facility?

- Yes
- No

1. If Yes, what is the imaging mode used at your facility?

- Pulsed
- Continuous
- High dose
- Others, Please specify _________

1. Are you aware of the pulse rate of VFSS recording system used at your facility?

- Yes
- No

1. If Yes, what is the pulse rate of VFSS recording system used at your facility?

- _________

1. Are you aware of the frame rate of VFSS recording system used at your facility?

- Yes
- No

1. If Yes, what is the frame rate of VFSS recording system used at your facility?

- _________

1. Do you follow radiation safety measures during the VFSS procedure at your facility?

- Yes
- No

1. If Yes, what specific safety measures do you employ?

- _________

**Protocol and assessment method**

1. Do you use any specific VFSS assessment protocol at your facility?

Please specify _________

1. What type of consistencies are assessed during the VFSS recording? Select as many.

- Liquids only
- Semi solids only
- Solids
- Other modified consistencies, Please specify _________

1. Are you aware of consistencies modification standards such as the IDDSI, NDI etc?

- Yes
- No

1. Do you modify consistencies based on the IDDSI or any other international standards?

- Yes
- No

1. What contrast material is used for VFSS studies at your setup?

- Barium sulphate liquid
- Barium sulphate powder
- Others, Please specify _________

1. Are you aware of the concentration of barium sulphate used during VFSS recording?

- Yes
- No

1. If Yes, what specific concentration of barium sulphate do you use?

- Please specify _________

1. Do you screen for oesphageal dysphagia during VFSS recording?

- Yes
- No

1. If NO, what is the reason for not screening oesophageal dysphagia during VFSS recording?

- Please specify _________

1. Who analyses VFSS recordings at your facility?

- I, singlehandedly analyse VFSS recordings.
- I analyse VFSS recordings along with radiologist/radiographer.
- VFSS recordings are analysed by radiologist/radiographer alone.
- Others, Please specify _________

1. Do you use any specific VFSS analysis protocol at your facility?

Please specify _________

1. Do you use any specific rating scale(s) to analyse VFSS recordings at your facility?

- Yes
- No

1. If Yes, what specific rating scale(s) are used to analyse VFSS recordings at your facility?

- Please specify _________

1. How could we improve the existing protocol or practice pattern? Please provide your suggestions below.
